# Longitudinal trajectories in negative symptoms and changes in brain cortical thickness: 10-year follow-up study

**DOI:** 10.1192/bjp.2022.192

**Published:** 2023-07

**Authors:** Manuel Canal-Rivero, Miguel Ruiz-Veguilla, Victor Ortiz-García de la Foz, Alvaro López-Díaz, Nathalia Garrido-Torres, Rosa Ayesa-Arriola, Javier Vazquez-Bourgon, Jacqueline Mayoral-van Son, Paolo Brambilla, Tilo Kircher, Rafael Romero-García, Benedicto Crespo-Facorro

**Affiliations:** Mental Health Service, Hospital Universitario Virgen del Rocío, Seville, Spain; Centro de Investigación Biomédica en Red de Salud Mental, Instituto de Salud Carlos III (CIBERSAM), Madrid, Spain; and Instituto de Biomedicina de Sevilla (IBiS)/HUVR/CSIC/Universidad de Sevilla, Seville, Spain; Mental Health Service, Hospital Universitario Virgen del Rocío, Seville, Spain; Centro de Investigación Biomédica en Red de Salud Mental, Instituto de Salud Carlos III (CIBERSAM), Madrid, Spain; Instituto de Biomedicina de Sevilla (IBiS), Seville, Spain; and Department of Psychiatry, Universidad de Sevilla, Seville, Spain; Department of Psychiatry, Marqués de Valdecilla University Hospital, Santander, Spain; Instituto de Investigación Sanitaria (IDIVAL), Santander, Spain; and School of Medicine, University of Cantabria, Santander, Spain; Hospital Universitario Virgen Macarena, Seville, Spain; and Centro de Investigación Biomédica en Red de Salud Mental, Instituto de Salud Carlos III (CIBERSAM), Madrid, Spain; Mental Health Service, Hospital Universitario Virgen del Rocío, Seville, Spain; and Centro de Investigación Biomédica en Red de Salud Mental, Instituto de Salud Carlos III (CIBERSAM), Madrid, Spain; Department of Pathophysiology and Transplantation, University of Milan, Milan, Italy; and Department of Neurosciences and Mental Health, Fondazione IRCCS Ca' Granda Ospedale Maggiore Policlinico, Milan, Italy; Department of Psychiatry and Psychotherapy, University of Marburg, Marburg, Germany; Instituto de Biomedicina de Sevilla (IBiS)/HUVR/CSIC/Universidad de Sevilla, Seville, Spain; Department of Medical Physiology and Biophysics, University of Seville, Seville, Spain; Centro de Investigación Biomédica en Red de Salud Mental, Instituto de Salud Carlos III (CIBERSAM), Madrid, Spain; and Department of Psychiatry, University of Cambridge, Cambridge, UK

**Keywords:** Negative symptoms, first episode psychosis, cortical thickness, factor analysis, MRI

## Abstract

**Background:**

Understanding the evolution of negative symptoms in first-episode psychosis (FEP) requires long-term longitudinal study designs that capture the progression of this condition and the associated brain changes.

**Aims:**

To explore the factors underlying negative symptoms and their association with long-term abnormal brain trajectories.

**Method:**

We followed up 357 people with FEP over a 10-year period. Factor analyses were conducted to explore negative symptom dimensionality. Latent growth mixture modelling (LGMM) was used to identify the latent classes. Analysis of variance (ANOVA) was conducted to investigate developmental trajectories of cortical thickness. Finally, the resulting ANOVA maps were correlated with a wide set of regional molecular profiles derived from public databases.

**Results:**

Three trajectories (stable, decreasing and increasing) were found in each of the three factors (expressivity, experiential and attention) identified by the factor analyses. Patients with an increasing trajectory in the expressivity factor showed cortical thinning in caudal middle frontal, pars triangularis, rostral middle frontal and superior frontal regions from the third to the tenth year after the onset of the psychotic disorder. The *F*-statistic map of cortical thickness expressivity differences was associated with a receptor density map derived from positron emission tomography data.

**Conclusions:**

Stable and decreasing were the most common trajectories. Additionally, cortical thickness abnormalities found at relatively late stages of FEP onset could be exploited as a biomarker of poor symptom outcome in the expressivity dimension. Finally, the brain areas with less density of receptors spatially overlap areas that discriminate the trajectories of the expressivity dimension.

As recognised in DSM-5, negative symptoms are one of the core symptoms of schizophrenia.^1,2^ Despite the clinical relevance of these symptoms, their psychopathological dimensions in people with first-episode psychosis (FEP) have not been adequately identified.^3^ This is important, because negative symptoms have been shown to better predict psychosocial functioning.^4^

A few studies have explored potential trajectories of negative symptoms in people with FEP by combining data covering extensive follow-up periods with statistical approaches capable of coping with unobserved trajectories, such as the latent growth mixture modelling (LGMM). LGMM has been a progress in trajectory analysis since this approach allows identification of classes of individuals characterised by different multivariate normal distribution. Moreover, LGMM uses robust maximum likelihood estimation, which can accommodate missing data.^5^ The identification of these trajectories as well as the development of potential predictors of poor prognosis could contribute to the implementation of therapeutic policies for reducing the burden of psychosis on quality of life.^6^

The identification of potential structural brain abnormalities associated with different trajectories of negative symptoms could complementarily contribute to the discovery of predisposing factors, which may improve prediction accuracy. An extensive literature has described a relationship between negative symptoms and decreased grey matter in people at clinical high risk for psychosis.^7^ Small sample sizes and, more importantly, the lack of long-term follow-up of clinical assessments and neuroimaging data have limited the potential of neuroimaging biomarkers in supporting therapeutic interventions that can minimise the side-effects of negative symptoms.

The molecular mechanisms driving cortical abnormalities in psychiatric conditions are still poorly understood. In this line, recent studies reveal that micro-architectural markers related to metabolism, cellular components and neurotransmitter receptors/transporters are associated with abnormalities in brain structure across disorders,^8^ suggesting that molecular vulnerability drives cortical disorder profiles.^9–11^ Nevertheless, the associations of these markers with long-term/longitudinal brain changes in psychosis remains unexplored.

The main aim of the present study was to explore potential brain abnormalities related to different trajectories of negative symptom evolution in FEP over a 10-year follow-up period. Secondarily, we aimed to analyse potential associations between resulting cortical thickness profiles and the spatial map of six molecular predictors (gene expression gradient, receptor density, excitatory/inhibitory ratio, glycolytic index, glucose metabolism, synapse density).

## Method

### Participants

Data for the current study were obtained from a large cohort of patients representative of the general population of individuals experiencing an FEP in the epidemiological catchment area comprising the autonomous community of Cantabria, located on the northern coast of Spain.^12^ The patients were being treated in a longitudinal intervention programme (Programa de Atención a Fases Iniciales de Psicosis [Early Intervention Programme for First Episode Psychosis] (PAFIP)) conducted at the University Hospital Marqués de Valdecilla, Santander, Spain.^12–14^ A total of 357 individuals with FEP were recruited for this study (see Supplementary Material, available at https://dx.doi.org/10.1192/bjp.2022.192).

### Ethics

The authors assert that all procedures contributing to this work comply with the ethical standards of the relevant national and institutional committees on human experimentation and with the Helsinki Declaration of 1975, as revised in 2008. The study was approved by the ethics committee for clinical research, CEIC Cantabria, in accordance with international standards for research ethics (clinical trial numbers NCT0235832 and NCT02534363). Patients who met the criteria and provided written informed consent, along with their families, were entered into PAFIP and PAFIP-10 for reassessment. This study followed the Strengthening the Reporting of Observational Studies in Epidemiology (STROBE) reporting guideline for cohort studies.^15^

### Demographic and clinical assessments

Sociodemographic information was recorded from interviews with patients, their relatives and from medical records on admission. This included information regarding years of education, socioeconomic status derived from the parents’ occupation (‘low qualification worker’ versus ‘other’), relationship status (‘married/cohabiting’ versus ‘single/divorced/separate or widowed’), employment status (‘employed’ versus ‘unemployed’) and first-degree family history of psychosis, which was based on participant and family reports (‘yes’ versus ‘no’).

Age at psychosis onset, duration of untreated illness (DUI) and duration of untreated psychosis (DUP) were obtained. Social functioning was assessed at baseline using the Disability Assessment Schedule Spanish version.^16^ Depression was assessed by the Calgary Depression Scale for Schizophrenia (the higher the score, the more depressive symptoms).^17^ The Simpson–Angus Scale was used to assess Parkinsonian movement disorder.^18^ Mean antipsychotic doses, expressed as chlorpromazine equivalents (CPZeq),^19^ were as follows: olanzapine 5–20 mg/day (100–400 CPZeq), risperidone 3–6 mg/day (150–300 CPZeq), haloperidol 3–9 mg/day (150–450 CPZeq), quetiapine 100–600 mg/day (133.33–800 CPZeq), ziprasidone 40–160 mg/day (66.67–266.67 CPZeq) and aripiprazole 5–30 mg/day (66.67–400 CPZeq).

The neuropsychological battery was administered by trained neuropsychologists. A subset of measures was selected to assess eight cognitive areas. In addition, a composite metric known as global cognitive functioning (GCF) was obtained using seven cognitive domains evaluated (verbal memory, visual memory, executive functioning, working memory, processing speed, motor dexterity and attention). Further details are given in the Supplementary Material.

Clinical negative symptoms of psychosis were assessed using the Scale for the Assessment of Negative Symptoms (SANS).^20^ An experienced psychiatrist (B.C.-F.) carried out all negative symptom assessments using the SANS throughout the follow-up period.

Clinical follow-up assessments were obtained at six different time points: baseline, understood as the point at which participants were included in the PAFIP programme, and at 6 weeks, 3 months, 1 year, 3 years and 10 year after programme entry. Change scores for negative symptoms, depressive symptoms, Simpson–Angus Scale, GCF and chlorpromazine equivalent doses, as well as changes in the dimensions obtained in the factor analyses of SANS, were calculated. Change scores were calculated as the score at baseline minus the score at 10-year follow-up (Table 1).

**Table 1:** Sociodemographic and clinical characteristics

	Baseline (*n* = 322)	6-week follow-up (*n* = 285)	3-month follow-up (*n* = 213)	1-year follow-up (*n* = 281)	3-year follow-up (*n* = 248)	10-year follow-up (*n* = 111)
Age, years: mean (s.d.)	29.7 (8.79)					
Gender (male), *n* (%)	217 (60.8%)					
Education, years: mean (s.d.)	10.23 (3.28)					
Family history of psychosis, *n* (%)	76 (21.3%)					
Low socioeconomic status, *n* (%)	176 (49.3%)					
Unmarried, *n* (%)	265 (74.2%)					
Unemployed, *n* (%)	145 (40.6%)					
DUI, months: mean (s.d.)	19.46 (34.9)					
DUP, months: mean (s.d.)	10.4 (22.99)					
SANS total, mean (s.d.)	12.03 (13.86)	7.21 (10.13)	6.60 (9.22)	7.18 (12.21)	4.28 (9.05)	10.47 (14.95)
SAPS total, mean (s.d.)	26.28 (12.19)	4.53 (7.32)	1.93 (5.15)	1.62 (5.01)	0.99 (3.06)	1.88 (5.72)
Positive symptoms, mean (s.d.)	7.33 (2.46)	2.10 (2.52)	0.95 (1.99)	0.71 (1.80)	0.76 (1.8)	0.93 (1.99)
Negative symptoms, mean (s.d.)	4.14 (5.13)	3.71 (4.46)	3.89 (4.47)	3.65 (4.73)	3.12 (4.84)	4.38 (4.96)
Disorganised symptoms, mean (s.d.)	6.62 (3.54)	1.25 (2.30)	0.53 (1.56)	0.36 (1.27)	0.45 (1.58)	0.50 (1.67)
Change in SANS score between baseline and final follow-up period, mean (s.d.)						5.01 (17.70)
Change in SAPS score between baseline and final follow-up period, mean (s.d.)						25.51 (12.34)
DAS, mean (s.d.)	1.45 (1.51)		1.50 (1.28)	1.22 (1.35)	1.09 (1.37)	0.98 (1.14)
CDSS, mean (s.d.)	2.25 (3.19)	1.43 (2.77)	1.58 (3.13)	0.60 (1.56)	0.54 (1.63)	0.87 (2.34)
Change in CDSS score between baseline and final follow-up period, mean (s.d.)						1.64 (4.27)
Simpson–Angus Scale, mean (s.d.)	0.42 (1.06)	1.11 (2.19)	0.89 (2.02)	0.04 (1.10)	0.44 (1.34)	0.82 (1.57)
Change in Simpson–Angus score between baseline and final follow-up period, mean (s.d.)						−0.25 (2.10)
Antipsychotic dose, CPZeq: mean (s.d.)	194.72 (81.11)	389.97 (159.45)	323.63 (156.70)	239.45 (168.54)	255.01 (194.93)	394.97 (212.08)
Change in antipsychotic dose, CPZeq: mean (s.d.)						
Attention, mean (s.d.)	−2.08 (3.76)			−1.75 (3.85)	−1.76 (3.85)	−1.71 (4.14)
Verbal memory, mean (s.d.)	−2.35 (1.38)			−1.98 (1.49)	−2.01 (1.46)	−1.99 (1.38)
Visual memory, mean (s.d.)	−0.54 (0.98)			−0.01 (1.06)	−0.37 (0.96)	−0.62 (0.87)
Processing speed, mean (s.d.)	−1.41 (1.11)			−0.90 (1.26)	−1.03 (1.20)	−0.68 (1.11)
Working memory, mean (s.d.)	−0.48 (0.88)			−0.36 (0.88)	−0.47 (0.82)	−0.54 (0.82)
Executive function, mean (s.d.)	−1.12 (1.94)			−0.84 (1.92)	−0.90 (2.25)	−0.82 (1.91)
Motor dexterity, mean (s.d.)	−1.11 (2.34)			−0.74 (2.38)	−0.68 (1.67)	−1.22 (2.64)
GCF, mean (s.d.)	1.33 (0.95)			1.06 (0.95)	1.04 (0.91)	1.04 (0.94)
Change in GCF score between baseline and final follow-up period, mean (s.d.)						0.39 (0.76)
Expressivity, mean (s.d.)	2.88 (5.38)	1.98 (4.02)	1.71 (3.67)	2.23 (4.89)	1.51 (4.09)	3.33 (5.35)
Experiential, mean (s.d.)	4.50 (7.18)	3.68 (5.54)	4.55 (6.76)	4.24 (6.62)	2.85 (5.60)	4.97 (7.33)
Attention (derived from SANS factor analyses), mean (s.d.)	3.32 (3.3)	1.03 (1.85)	0.69 (1.68)	0.48 (1.40)	0.55 (1.46)	0.56 (1.80)
Change in expressivity score between baseline and final follow-up period, mean (s.d.)						0.80 (7.63)
Change in experiential score between baseline and final follow-up period, mean (s.d.)						0.86 (7.96)
Change in attention score between baseline and final follow-up period, mean (s.d.)						3.03 (3.78)

DUI, duration of untreated illness; DUP, duration of untreated psychosis; SANS, Scale for the Assessment of Negative Symptoms; DAS, Disability Assessment Schedule; CDS, Calgary Depression Scale for Schizophrenia; CPZeq, chlorpromazine equivalents; GCF, global cognitive functioning.

### Statistical factor analyses

Statistical analyses were conducted using the Statistical Package for the Social Sciences (SPSS), version 25 for Windows.^21^ RStudio version 1.4.1106^22^ for Windows was also used for structural equation modelling analyses using the lavaan package^23^ and growth mixture modelling using the Latent Class Mixed Models package.^24^ Exploratory and confirmatory factor analyses are detailed in the Supplementary Material.

### Latent growth mixture modelling analyses

LGMM was used to determine the number of latent classes in each of the SANS dimensions identified by factor analyses. GMM is a classification technique in which each participant is included in only one class (latent class), and each class is characterised by a specific trajectory over time. Membership of a participant was determined by calculating the posterior probability of belonging to one class and assigning the patient to the class with the highest probability. Latent classes were formed based on scores on each of the SANS dimensions evaluated at the six time points over the 10-year follow-up period (baseline, 6 weeks, 3 months, 1 year, 3 years and 10 years). Missing data were omitted by default. The best-fitting model according to the goodness-of-fit indices of Akaike's information criterion (AIC), Bayesian information criterion (BIC), sample-size-adjusted BIC (aBIC) and entropy was used to determine the optimal number of trajectory classes, from a total of 4 possible trajectories. Lower AIC, BIC and aBIC values suggest a more parsimonious model, and higher entropy indicates a better model fit. Entropy ranges from 0 to 1; values approaching 1 indicate a clear delineation of classes. Aside from fit statistics, interpretability and parsimony of the model were also taken into consideration in model selection.

### Neuroimaging acquisition and pre-processing

Participants underwent magnetic resonance imaging (MRI) of the brain at baseline and at the 2-year, 3-year, 5-year and 10-year follow-up periods. All images were acquired using the same 3 T Philips Medical Systems MRI scanner (Achieva, Best, The Netherlands) using an 8-channel head coil at the Hospital Marques of Valdecilla. A 3D *T*_1_-weighted sequence was acquired with the following parameters: repetition time TR = 8.2 ms, echo time TE = 3.7 ms, flip angle 8°, acquisition matrix 256 × 256, voxel size 0.94 × 0.94 × 1 mm and 160 contiguous slices. Images were first visually inspected for artefacts and gross anatomical abnormalities. Pre-processing of MRI data was undertaken using Freesurfer pipelines, version 6.0. In brief, each image was subjected to skull stripping, segmentation and surface reconstruction.^25,26^ Cortical thickness measurements were estimated by reconstructing the pial surface and the boundary between grey matter and white matter and measuring the distance between these surfaces. Cortical thickness values were averaged across all vertices included in each of the 68 cortical parcels defined in the Desikan–Killiany atlas.^25^ Regional cortical thickness values were collapsed across hemispheres by computing the mean cortical thickness for left and right regional values, resulting in 34 cortical regions. Hemispheric-averaged regional values were (arbitrarily) plotted over the representation of the left hemisphere. The Euler index was computed for each individual as a proxy of image quality, which has shown a similar degree of accuracy to that of human raters.^27^

### Molecular cortical maps

Similarly to the methodology proposed by Hansen et al,^28^ we tested potential associations between resulting cortical thickness cortical profiles and the spatial map of the following six biological predictors derived elsewhere.

#### Gene expression gradient

The first principal component of gene expression data from the whole genome was used to represent the variation in expression levels across the left cortex. Data were collected by the Allen Human Brain Atlas.^29^ A detailed account of the specific processing choices made can be found in Hansen et al.^30^

#### Receptor density

The first principal component of receptor density was used to represent the variation in receptor densities across the cortex. The marker included positron emission tomography (PET) tracer studies for a total of 18 receptors and transporters, across 9 neurotransmitter systems (dopamine, noradrenaline, serotonin, acetylcholine, glutamate, gamma-aminobutyric acid (GABA), histamine, cannabinoid and opioid). Parcellated PET maps were then *z*-scored before compiling all receptors/transporters into a region × receptor matrix of relative densities. The data were originally presented as an atlas in Hansen et al.^31^

#### Excitatory/inhibitory ratio

The excitatory/inhibitory ratio was computed as the ratio of *z*-scored PET-derived excitatory (5-HT2_A_, 5-HT_4_, 5-HT_6_, D_1_, mGluR_5_, α_4_β_2_ and M_1_) to inhibitory (5-HT_1A_, 5-HT_1B_, CB_1_, D_2_, GABA_A_, H_3_ and μ-opioid) neurotransmitter receptor densities in the cortex, using the same data-set that was used to compute the receptor density.^28^

#### Glycolytic index

This index was calculated as the residual after linearly fitting glucose metabolism to oxygen metabolism, as described by Vaishnavi et al.^32^ Larger values indicate more aerobic glycolysis.

#### Glucose metabolism

Glucose metabolism in the cortex was measured in 33 healthy adults by administering ^18^F-labelled fluorodeoxyglucose (FDG) for a PET scan.^32^

#### Synapse density

Synapse density in the cortex was measured in 76 healthy adults by administering ^11^C-UCB-J, a PET tracer that binds to the synaptic vesicle glycoprotein 2A (SV2A).^33^

### Statistical analyses of imaging data

Prior to statistical assessment, we removed 19 participants with all their regions identified as outliers (defined as cortical thickness exceeding five standard deviations from the mean) and 13 regional outliers from 13 different individuals. Age, gender and total intracranial volume were regressed out from cortical thickness estimates using multiple regression and the residuals were added to the group mean to allow for easier interpretation. Traditional analysis of covariance (ANCOVA) using cortical thickness as the dependent variable and time (and SANS dimensions) as independent variables could not be conducted because the data violate the assumption of homoscedasticity (i.e. cortical thickness variance increases across follow-up time, Levene's test, *P* = 0.001). For this reason, longitudinal changes in cortical thickness were evaluated by subtracting long-term (at 10 years) from short-term (at 1, 2 and 3 years) cortical thickness.

Analysis of variance (ANOVA) implemented in MATLAB 2022a (MathWorks) was used to test for group effects (stable, decreasing and increasing) on cortical thickness changes for each of the SANS factors. The resulting *F*-statistic maps were associated with each of the seven molecular maps described above. The false discovery rate (FDR) was used to correct *P*-values (*P*_FDR_) for regional-based analyses and factors (i.e. 34 regions × 2 factors gives 64 statistical tests when testing across regions and 6 statistical tests when testing across molecular profiles).

## Results

### Sample characteristics

We included 357 participants with FEP in this study. Different manifestations of negative symptoms were evaluated at six different times over a 10-year follow-up period. Information about sociodemographic, clinical and neuropsychological characteristics of the sample is shown in Table 1. Exploratory and confirmatory factor results are detailed in Supplementary Figs 1 and 2.

A total of 260 (72.8%) and 119 participants (33.3%) completed a negative symptoms evaluation using the SANS at 3-year and 10-year follow-up respectively. A more parsimonious analysis including the six follow-up periods revealed that 11 participants (3.1%) were not evaluated at any of the follow-up periods included in the study and 249 (69.7%) were tested at at least one of the follow-ups. *T*-test analysis showed no significant differences in cortical thickness at baseline between those who completed the evaluation at 10-year follow-up and those who did not (*P* = 0.78).

### Evolution of SANS dimensions

**Fig. 1:**
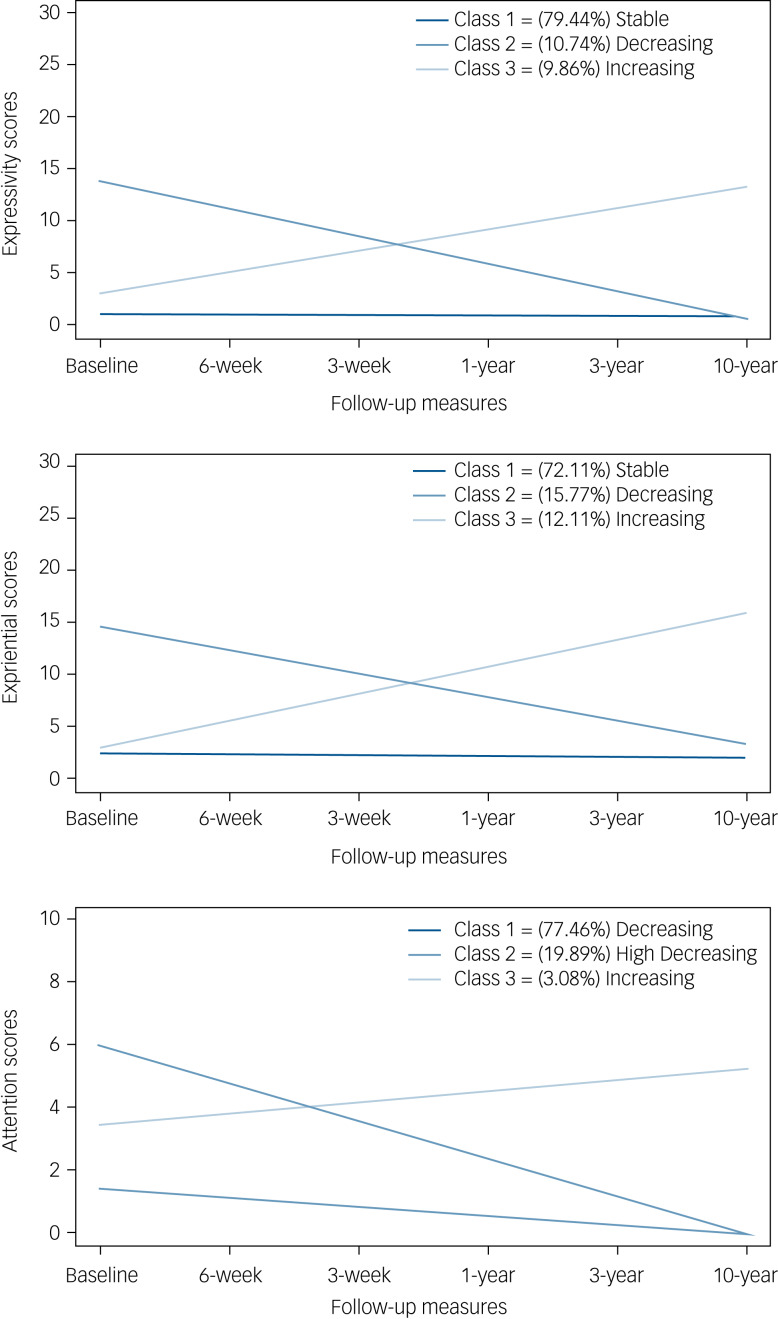
Trajectories (stable, decreasing and increasing) of the three negative symptom dimensions as grouped by the latent class mixed model.

The three-class GMM model was selected for each of the SANS dimensions identified after examining fit indices, entropy and parsimony (Table 2). The three mean trajectories estimated by the three-class model for each of the SANS dimensions (expressivity, experiential and attention) are depicted in Fig. 1.

**Table 2:** Latent class mixed model fit of the three SANS dimension trajectories

Number of classes	Number of parameters	Fit statistics				% of the sample in each class			
		AIC	BIC	aBIC	Entropy	Class 1	Class 2	Class 3	Class 4
Expressivity									
1	4	8476.66	8492.15	8479.46	1.00	100.00%			
2	8	8261.06	8292.04	8266.66	0.90	89.30%	10.70%		
3a	12	8027.25	8073.72	8035.65	0.90	79.44%	10.70%	9.86%	
4	16	8035.25	8097.20	8046.45	0.63	78.31%	10.99%	10.70%	0.01%
Experiential									
1	4	9711.55	9727.04	9714.35	1	100%			
2	8	9630.94	9661.92	9636.54	0.56	80.28%	19.71%		
3a	12	9550.71	9597.18	9559.11	0.75	72.11%	15.77%	12.11%	
4	16	9558.71	9620.67	9569.91	0.48	70.74%	16.62%	12.68%	0.01%
Attention									
1	4	7144.43	7159.94	7147.25	1	100%			
2	8	6977.83	7008.85	6983.47	0.72	78.15%	21.85%		
3a	12	6912.45	6958.99	6920.92	0.85	77.46%	19.89%	3.08%	
4	16	6920.45	6982.50	6931.74	0.80	76.50%	19.89%	3.36%	0.01%

SANS, Scale for the Assessment of Negative Symptoms; AIC, Akaike information criterion; BIC, Bayesian information criterion; aBIC, sample size-adjusted Bayesian information criterion.

a.The preferred model.

Most of the sample was included in a trajectory characterised by the presence of stable pattern during the follow-up period. The number of participants belonging to each trajectory identified for the expressivity factor were: 282 (79.44%) participants belonging to class 1 (‘stable’), 38 (10.74%) to class 2 (‘decreasing’) and 35 (9.86%) to class 3 (‘increasing’). For the second SANS dimension (experiential), 256 (72.11%) belonged to the ‘stable’ trajectory, 56 (15.77%) were included in the second class (‘decreasing’) and 43 (12.11%) in the third class (‘increasing’). Finally, the attention dimension was also formed of three trajectories, whose distribution was as follows: 275 (77.46%) in class 1 (‘decreasing’), 71 (19.89%) in class 2 (‘high decreasing’) and 11 (3.08%) in class 3 (‘increasing’).

### SANS factor associations with whole-brain cortical thickness

The attention factor was excluded from the neuroimaging analyses since the National Institute of Mental Health (NIMH) Measurement and Treatment Research to Improve Cognition in Schizophrenia (MATRICS) initiative has recently recommended that attention should not be included as negative symptom. On the other hand, the attention dimension was formed from the items that evaluate inattention in the original scale, and we agree with NIMH MATRICS that this dimension corresponds more to cognitive function than to negative symptoms.

**Fig. 2:**
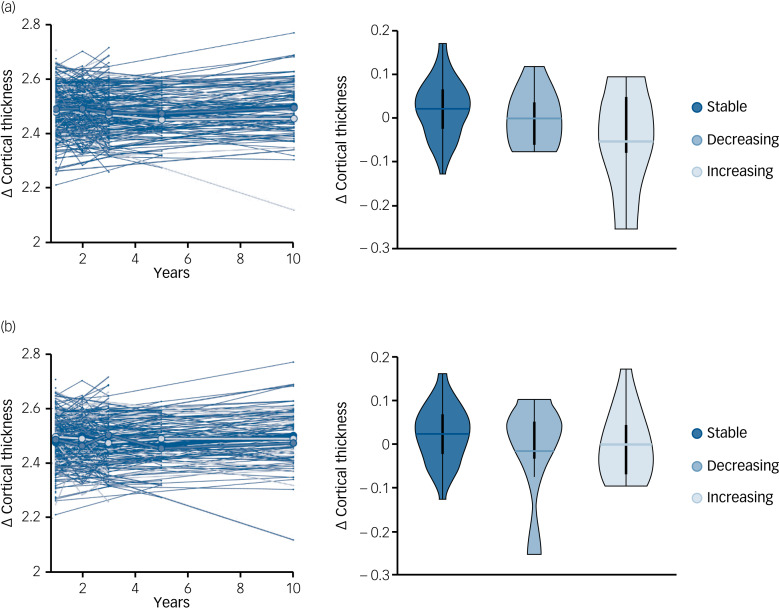
Whole-brain cortical thickness changes for experiential and expressivity factors on the Scale for the Assessment of Negative Symptoms (SANS). Longitudinal cortical thickness changes across time for expressivity (a) and experiential (b) factors, with box plots showing the long-term cortical thickness changes across factors.

The average Euler index of our cohort, a proxy of MRI quality, was −99.4 (s.d. = 89), similar to what has been reported in other cohorts.^34^ Age-, gender- and intracranial volume-corrected cortical thickness was longitudinally assessed at whole-brain level across SANS factors. Cortical thickness was similar across trajectories (stable, decreasing, increasing) during the first 3 years after FEP symptoms manifested (Fig. 2). However, the ANOVA analysis revealed a significant group effect when comparing long-term cortical thickness changes (calculated as long-term cortical thickness (at 10 years) minus short-term cortical thickness (at 1, 2 and 3 years)) for expressivity (*P* = 0.005) but not experiential (*P* = 0.13) factors. *Post hoc* analyses revealed a significant difference between the stable and increasing groups (*P* = 0.0018) but not between the stable and decreasing (*P* = 0.25) or decreasing and increasing (*P* = 0.20) groups. The impact of clinical covariates on longitudinal changes in cortical thickness was also tested. We found no significant association between cortical thickness changes and global cognitive functioning, positive symptoms, disorganised symptoms or attrition (Supplementary Fig. 3). However, chlorpromazine-equivalent doses were significantly associated with cortical thickness changes (*P* < 10^−4^). Consequently, the main analyses were repeated after correcting for medication (Supplementary Fig. 4).

### Regional cortical thickness associations with SANS factors

**Fig. 3:**
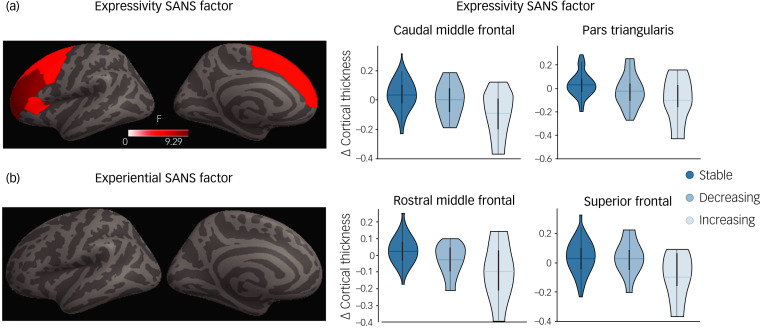
Regional long-term cortical thickness changes for expressivity and experiential factors on the Scale for the Assessment of Negative Symptoms (SANS). Regions showing significant group effects (stable, decreasing, increasing trajectories) in long-term cortical thickness changes for expressivity (a) and experiential (b) factors. The scatter plots of longitudinal cortical thickness changes by region show a significant group effect for expressivity.

Long-term changes in cortical thickness were assessed across dimensions at regional level. Four regions (caudal middle frontal, pars triangularis, rostral middle frontal and superior frontal) showed a significant group effect in cortical thickness across the stable, decreasing and increasing trajectories for the expressivity factors (*P*_FDR_ < 0.05; Fig. 3(a)). These four regions showed similar short-term cortical thickness values, but they diverge at 10 years after FEP diagnosis (Fig. 3(b)). These results were replicated after correcting for medication (Supplementary Fig. 5). On the contrary, experiential dimensions did not show significant differences on cortical thickness that survived FDR correction (Fig. 3(a)).

### Expressivity group-effect on cortical thickness is associated with receptors density

**Fig. 4:**
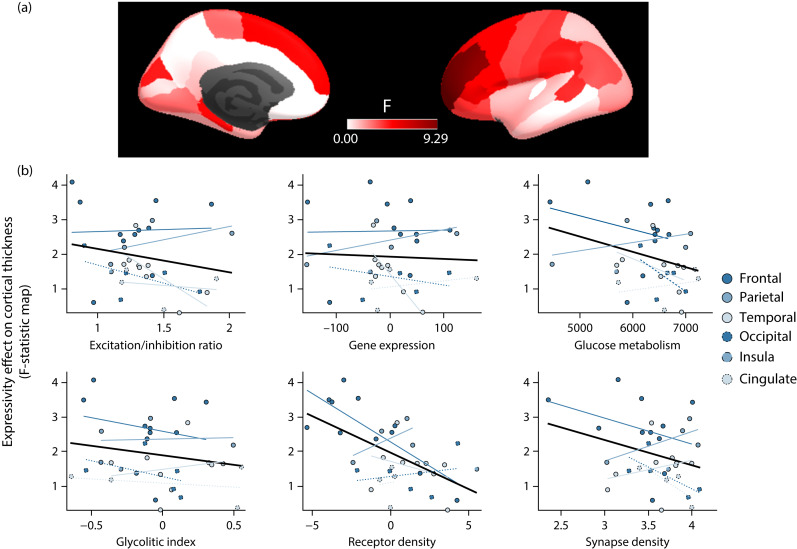
Association between differential cortical thickness trajectories across expressivity dimensions and molecular cortical profiles. (a) Cortical map showing the statistic (*F*-value) associated with the differential cortical thickness trajectories across the three expressivity dimensions. (b) Associations between differential cortical thickness trajectories across expressivity and each of the six molecular cortical maps considered here.

We tested whether the (unthresholded) *F*-statistic map of differential cortical thickness trajectories across the three expressivity trajectories described above was associated with a wide set of molecular cortical profiles (Fig. 4). None of the six maps considered showed a significant association except for the receptor density (*P*_FDR_ = 0.03). Thus, regions showing stronger group effect of expressivity on cortical thickness (i.e. higher *F*-values) had lower receptor density scores on the cortical map constructed by performing a principal component analysis of PET-derived maps for dopamine, noradrenaline, serotonin, acetylcholine, glutamate, GABA, histamine, cannabinoid and opioid receptors.

## Discussion

Here we present the first study using mixed model regression analysis to investigate 10 years of developmental trajectories of cortical thickness in relation to SANS dimensions in people with FEP. The main findings derived from our work were: (a) trajectory analyses displayed three different patterns in the psychopathological SANS dimensions differentiated by factor analyses, (b) participants who were characterised by an increasing trajectory in the expressivity dimension showed cortical thinning in caudal middle frontal, pars triangularis, rostral middle frontal and superior frontal regions from the third year to the tenth year after FEP onset and (c) the areas with less density of receptors (dopamine, noradrenaline, serotonin, acetylcholine, glutamate, GABA, histamine, cannabinoid and opioid) spatially overlap brain areas that discriminate the trajectories of the expressivity dimension.

Our results reflected three different dimensions on the SANS (i.e. expressivity, experiential and attention). Previous factor analyses of negative symptoms in people with FEP have not included attentional items, which would explain the absence of the attentional dimension in these studies.^35^ The attentional dimension reported in our study reflects the inclusion of those items contained in the original version of the scale. Our results highlight that the dimension of inattention is independent of the expressivity and experiential dimensions and is formed from the items originally designed to assess inattention in the original SANS. Excluding attention, our data align with those of Kirkpatrick et al, who suggested that negative symptoms can be grouped into two domains: avolition/apathy and diminished expression.^36^

Trajectory analyses have been proposed as a fundamental tool not only for understanding the longitudinal course patterns of negative symptoms but also for implementing early intervention programmes that facilitate patients’ recovery from FEP.^6^ Previous studies that have considered the same follow-up period have also reported the same three trajectories (i.e. stable, decreasing and increasing).^37,38^ Most of our participants showed stable evolution of negative symptoms or decreasing response, consistent with previous studies using similar follow-up periods.^37^ It is worth noticing that Austin et al reported a fourth trajectory, identified as no response, which was formed by 27% of their sample.^38^ As pointed out by the authors, those who received standard treatment presented worse negative course than those included in assertive treatment. Following this reasoning, the lack of a non-responder group in our data-set may be due to the interventions carried out in the PAFIP programme. PAFIP is a multidisciplinary programme combining a set of pharmaceutical, psychological and community interventions with the aim of improving patients’ functionality. Thus, our findings would indicate that the implementation of specific early intervention programmes may have an important effect on the evolution of negative symptoms. As recently argued by Chang et al, assessing symptoms in drug-naive patients and performing early follow-up evaluation may contribute to the identification of responders.^6^ Baseline assessments dominated by acute phases rather than first episodes may be the reason why some findings diverge across studies.^38^

In our study, we observed significant differences in longitudinal cortical thickness changes across recovery groups (stable, decreasing and increasing) for the expressivity but not for the experiential dimension. Participants with increasing expressivity showed greater cortical thinning between 3 and 10 years after the onset of the psychotic disorder in four particular regions (i.e. caudal middle frontal, pars triangularis, rostral middle frontal and superior frontal).

Prefrontal thinning and negative symptoms have been closely related.^39^ As mentioned above, expressivity was formed mainly by items evaluating blunted affect. Blunted affect has been defined as difficulty in expressing emotions, characterised by diminished facial expression, expressive gestures and vocal expressions in reaction to emotion-provoking stimuli. The pars triangularis is involved in semantic processing of language as well as in non-verbal communication such as gesticulation, facial expression and modulation of timing and intonation of speech, which would explain deficits in blunted affect.^40^ Other regions, such as the caudal and rostral middle frontal gyrus and superior frontal gyrus, have also shown increasing reduction from 3 to 10 years after FEP onset. Middle frontal gyrus and superior frontal gyrus regions have been closely related to higher cognitive functions, executive function deficit and difficulties in the regulation of emotions.^41^ Greater thinning in these areas may explain deficits in motivation and goal-directed behaviour shown by the ‘decreasing’ group over the follow-up period.^42^ In contradiction to previous studies, we have failed to find brain alteration in the experiential dimension. This may be explained by differences in study design from previous literature (longitudinal versus cross-sectional).

An intriguing result from our study is that differences in cortical thickness started at the third year after FEP onset but not previously. This could be interpreted as a potential biomarker of persistence of negative symptoms in the expressivity dimension. This result highlights the importance of monitoring potential brain abnormalities over time to identify individuals at risk of worsening negative symptoms or even persistent symptoms in the expressivity dimension. Further studies are required to determine whether cortical thinning is linked to individual risks of negative symptoms or whether it is a side-effect of medication.

Kraepelin and Bleuler described the negative symptoms of schizophrenia as the absence or diminution of normal behaviours and functions. In particular, Kraepelin coined the term ‘avolitional syndrome’, which is understood as the foundation for the concept of the deficit syndrome in schizophrenia.^43^ Carpenter et al defined deficit syndrome as the presence of primary negative symptoms that are characterised by enduring traits and are also present between episodes of exacerbation of positive symptoms.^44^ In our study, we identified a group of individuals with FEP who are characterised by a progressive increase in the expressivity and experiential dimensions who could represent a pool of FEP patients with deficit syndrome or persistent negative symptoms. The identification of this group of patients and the analyses of potential predictors of poor negative symptom prognosis seems to be of vital importance for the implementation of therapeutic interventions for prevention and functionality improvements.

Bearing in mind that the dopamine hypothesis proposes that the negative symptoms are related to a dopaminergic hypofunction in the frontal lobe,^45^ it is interesting to note that we found that the areas with lower density of receptors (dopamine, noradrenaline, serotonin, acetylcholine, glutamate, GABA, histamine, cannabinoid and opioid) detected in controls^28^ coincide with the brain areas that discriminate the trajectories of the expressivity dimension. Based on the regional vulnerability hypothesis described in recent studies, this may suggest that disruption of frontal regions may be important for the onset of negative symptoms.

### Limitations and strengths

Some limitations must be considered. First, attrition may have biased the proportions in the ‘stable’, ‘decreasing’ and ‘increasing’ groups of participants. However, LGMM is a statistical tool robust to missing data. Second, six differentially spaced follow-up measurements of SANS were carried out over the follow-up period (baseline, 6 weeks, 3 months, 1 year, 3 years and 10 years), which would make it difficult to capture the dynamism of negative symptoms. A strength of the study was that factor analyses were conducted for different time points, improving the reliability of the results.

### Implications

Our finding that the SANS can be reduced to three factors (expressivity, experiential and attention) opens the possibility of developing predictive models that can anticipate poor outcomes. The identification of these factors could facilitate the implementation of therapeutic interventions for mitigating the progression of negative symptoms, including cognitive–behavioural therapy and cognitive remediation, which have been demonstrated to improve symptoms.^46,47^ Finally, cortical thickness abnormalities found at relatively late stages of FEP onset could be interpreted as specific biomarkers of poor evolution or persistent symptoms in the expressivity dimension that are linked to receptor density. Further analyses are required to confirm these results.

## Data Availability

Data are available from the corresponding author on request.

## Supplementary material

For supplementary material accompanying this paper visit https://doi.org/10.1192/bjp.2022.192. click here to view supplementary material
