# Supplementary material for: Longitudinal trajectories in negative symptoms and changes in brain cortical thickness: 10-year follow-up study
Source: Br J Psychiatry. 2023 Jul;223(1):309–18. doi: 10.1192/bjp.2022.192 (PMC10331319; doi:10.1192/bjp.2022.192)
Supplement: Supplementary file 1 [file bjpsup.zip › S0007125022001921sup001.docx]

# Accelerated frontal thinning associated with worsening negative symptomatology in First Episode Psychosis: A 10-year follow-up study.

SUPPLEMENTARY MATERIAL

Manuel Canal-Rivero^a,b,c^*., Miguel Ruiz-Veguilla^a,b,c,d^***.**, Ortiz-García de la Foz, V^e^., Alvaro López-Díaz ^b,c,f^., Nathalia Garrido-Torres ^a,b,c^., Rosa Ayesa-Arriola ^b,e^., Javier Vazquez-Bourgon ^b,e^., Jacqueline Mayoral van Son ^a,b^., Paolo Brambilla ^g^., Tilo Kircher ^h^., Rafael Romero-García^b,c,d,i^******^&^**.**, Benedicto Crespo-Facorro^a,b,c,d&^.

^a^ Mental Health Service, Hospital Universitario Virgen del Rocío, Sevilla, Spain.

^b^ Centro Investigación Biomédica en Red Salud Mental, CIBERSAM, Madrid, Spain.

^c^ Instituto de Biomedicina de Sevilla (IBiS) HUVR/CSIC/Universidad de Sevilla

^d^ Department of Psychiatry, Universidad de Sevilla, Spain.

^e^ Department of Psychiatry. Marqués de Valdecilla University Hospital, IDIVAL, School of Medicine, University of Cantabria, Santander, Spain

^e^ Department of Psychiatry, University of Cambridge, UK

^f^ Mental Health Service, Hospital Universitario Virgen Macarena, Sevilla, Spain.

^g^ University of Milan, Milan, Italy.

^h^Department of Psychiatry and Psychotherapy, University of Marburg, Marburg, Germany.

^i^ Department of Medical Physiology and Biophysics, University of Seville, Spain

* These authors should be considered joint first author

^&^ These authors should be considered joint senior author

**Corresponding author.

## Setting

Data for the current study were obtained from a large cohort of patients representative of the general population of individuals suffering from an FEP in an epidemiological catchment area, which is the autonomous community of Cantabria, located in the Northern coast of Spain ^1^. FEP patients were treated in a longitudinal intervention program (Programa de Atención a Fases Iniciales de Psicosis, PAFIP) conducted at the University Hospital Marqués de Valdecilla ^1–3^. Referrals to the PAFIP came from the inpatient unit and emergency room, outreach mental health services and from other health-care workers throughout the region of Cantabria. After the initial contact by a qualified psychiatric nurse, all participants were initially screened for the presence of psychotic symptoms and all diagnoses were made by an experienced psychiatrist (BC-F) using the Structured Clinical Interview for DSM-IV Axis I Disorders ^4^ after 6 months of the baseline visit and confirmed the presence of schizophrenia and other primary psychotic disorders (F20–F29). There were no additional exclusion criteria for MRI except those specific to scanning logistics (e.g., claustrophobia, braces). PAFIP includes inpatient and outpatient care and provides multidisciplinary (psychiatric nursing, psychology, psychiatry and social work) and specific and personalized clinical attention from the first contact with PAFIP staff up to 3 years. It has a strong track record with FEP subjects that has included a longitudinal component with multiple assessments. Considering that the PAFIP constitutes the only alternative form of mental health care for FEP, we could defend that the included sample constitutes an epidemiological representation of the population of patients with FEP occurring in the entire region of Cantabria. Accordingly, the patients included in the PAFIP reached an age-corrected (16–60) incidence rate for SDD of 1.38 per 10 000, a figure that is equivalent to the one reported in most epidemiological studies ^2^. PAFIP-10 is a long-term follow-up study at 10 years (range between 8 and 12 years) of individuals with an FEP who were initially included in PAFIP from February 2001 to July 2008. All patients included in the referred period were invited for a reassessment, which comprised the 10-year follow-up study group.

## Intervention program

All referrals to PAFIP were screened against the following inclusion criteria: were 15–60 years of age; lived in the catchment area; experienced their first episode of psychosis; and were antipsychotic medication naïve, or if previously treated, a total lifetime of adequate antipsychotic treatment of less than 6 weeks. Meeting the DSM-IV criteria for drug or alcohol dependence, having an intellectual disability and having a history of neurological disease or head injury were exclusion criteria. The diagnoses were confirmed through the use of the Structured Clinical Interview for DSM-IV (SCID-I) ^5^ conducted by an experienced psychiatrist within 6 months of the baseline visit.

All participants received 3 years of evidence-based phase specific individual interventions: pharmacotherapy (the lowest effective dose of antipsychotic as maintenance dosage, monitoring for clinical response and side effects, such as prevent weight gain), psychoeducation, addictions’ treatment, vocational and education plans, group interventions for patients and their families, as well as crisis intervention services, main components of FEP services ^6^ tracking the process of outcome with the expectation of a full recovery.

## Neuropsychological assessment

The neuropsychological battery was administered by trained neuropsychologists between week 6 and week 13, a period that seems to be the most appropriate to implement baseline assessment for neurocognitive studies ^7^ free of biases associated with an acute psychotic mental state. A subset of measures was selected to asses eight cognitive areas: (1) verbal memory was assessed with the Rey Auditory Verbal Learning Test (RAVLT) ^8^, delayed recall; (2) visual memory was assessed with the Rey Complex Figure (RCF) ^9^, delayed reproduction; (3) executive functioning was evaluated with the Trail Making Test (TMT) ^10^ time to complete TMT-B minus TMT-A; (4) working memory was measured by the WAIS-III Backward Digits scale, total subscore ^11^; (5) processing speed was assessed with the WAIS-III Digit Symbol subtest, standard total score ^11^; (6) motor dexterity was estimated with the Grooved Pegboard Handedness (GP) ^12^, time to complete with dominant hand; (7) attention was appraised with the Continuous Performance Test (CPT) , total number of correct responses; and (8) premorbid IQ was determined using the WAIS-III Vocabulary subtest ^13^, standard total score. In addition, a composite metric known as GCF was obtained using seven of the cognitive domains evaluated (verbal memory, visual memory, executive functioning, working memory, processing speed, motor dexterity and attention). This index was calculated using the deviation of the patients from the controls in each cognitive domain at baseline, 1-year 3-year and 10-year ^14^. Higher scores of GCF indicated poorer cognitive functioning.

## Factor Analyses

The Kolmogorov–Smirnov test examined the normality of variable distribution. The first aim of the study was to analyse the number of factors that best define the SANS. For this purpose, parallel analysis was initially conducted to confirm the number of factors. In particular, rawpar.sps script developed by O’Connor ^15^ was used to performed these analyses. Moreover, Exploratory Factor Analyses (EFA) were conducted to analyse the factor solution. Before EFA, the Kaiser-Meyer-Olkin (KMO) test and Bartlett’s test of sphericity were conducted to evaluate the factorability. As the KMO measure of sampling was adequate and the significance of Bartlett’s test of sphericity was less than 0.05, EFAs proceeded. Extracted factors were rotated by varimax rotation. For selection of items corresponding to each of the factors, item factor loadings must be greater than 0.50, and at least double its loading on the other factor ^16,17^. The reliability of items in each factor was examined by Cronbach’s α. EFA analyses were carried out with the scores obtained from the participants at baseline.

Confirmatory Factor Analyses (CFA) were also conducted. CFA analyses were made with SANS ratings obtained at 1-year after the inclusion of the patients in PAFIP program. Several model fit indices were used to examine the goodness-of-fit of the model with the given dataset: Root Mean Square Error of Approximation (RMSEA), Standardized Root Mean Squared Residual (SRMR), comparative fit index (CFI), and the Tucker-Lewis Index (TLI). CFA analyses were carried out with the scores obtained from the participants at 12-month after the onset of the psychotic disorder.

## Factor Results

### Exploratory Factor Results

The Kolmogorov-Smirnov test was statistically significant (p<.001) for all SANS items, rejecting the null hypothesis of normally distributed scores. A total of 20 items of SANS were included in the analyses. The items which evaluate globally each of the five different original dimensions of SANS were not included in the analyses. The number of factors was first explored using parallel analysis. The comparison between eigenvalues of the source data and eigenvalues of random data sets, suggested a retention of three factors. In relation to EFA, KMO revealed good sampling adequacy (KMO = .90), and Bartlett’s test of sphericity was 3577.24 (p<.001), supporting the implementation of the exploratory factor analysis. The three-factor solution accounted for 60.28% of the variance.

The analyses of the factor loadings revealed that items of poverty of speech, poverty of content of speech, blocking, increased latency of response and grooming and hygiene did not load twice as high on the factor with which they showed the strongest relationship. Therefore, the EFA was newly executed removing the items previously mentioned from the new analyses.

Item’s exclusion improved the EFA. In fact, explained variance increased from 60.28% to 65.51%. KMO and Bartlett´s test supported the exploratory analyses and all the items showed adequate factor loadings. **Table 1S** shows the loading factor associated to each item. Eigenvalues derived from original and random data using parallel analysis also revealed a three-factor solution (**Figure 1S**). In particular, SANS items which conformed each of the factors found were as follows: experiential by impersistence at work or school, physical anergia, recreational interest and activities, sexual activity, ability to feel intimacy and closeness and relationship with friends and peers. On the other hand, expressivity dimension was composed by unchanging facial expression, decreased spontaneous movements, paucity of expressive gestures, poor eye contact and lack of vocal inflections. Finally, attention was formed by inappropriate affect, social inattentiveness and inattentiveness during mental status testing.

Cronbach´s alpha coefficient was calculated to estimate internal consistency of the subscales, revealing reliability values of 0.89, 0.91 and 0.51 for experiential, expressivity, and attention, respectively.

### Confirmatory Factor Results

CFA was performed with the scores obtained at 1-year after the inclusion in PAFIP program. Initial results showed a non-optimal RMSEA (0.15) and SRMR (0.11) estimations. Given that, modification indices were used to identify possible discrepancies between the proposed and estimated model. These indices indicated a potential significant correlation between the uniqueness’s of items 5 and 7, 13 and 14, 14 and 17, 1 and 2, 17 and 18, 2 and 7, 2 and 3, 2 and 5, 1 and 15, 1 and 7, 6 and 17, 2 and 15, 4 and 5, 3 and 17, 5 and 18, 16 and 17, 7 and 19, experiential dimension and items 1, 5, 7, 19 as well as with 20. Finally, between expressivity dimension and items 13 and 20. CFA was repeated but this time excluding the correlation between the items previously mentioned. This time, fit indices showed good model fit: RMSEA=.06; SRMR=.04; CFI=.98; TLI = .97. The standardized factorial coefficients of the model are shown in **Figure 2S**.

## Discussion Factor Solution

Our results reflected three different dimensions from SANS (i.e., expressivity, experiential and attention). From our point of view, the attentional dimension found in our study responded to the inclusion of inattentional items included in the original version of SANS. However, we are in agreement that inattention should not be included into negative symptomatology as it responds to aspects more related to cognitive competences ^18^. It is worth to note that according to NIMH MATRICS attention has not be included into the negative symptomatology ^19^. Inappropriate affect should not belong to inattention factor from a theoretical point of view. However, statistical factor analyses revealed that this item seems to be part of the factor that we have named attention. It is worth to note that inappropriate affect have emerged as inconsistent item across different factor analyses studies ^20^. Our results are in line with those which have highlighted that the factor previously mentioned is characterized by inconsistences in their factor classification.

Expressivity dimension could be understood as blunted affect and alogia, encompassing a wide range of symptoms such as reduction of motivation and goal-directed behaviours. In our analyses, expressivity dimension was formed by the items included in affective blunting. These results align with previous studies ^21^. However, and contrary to our expectations, those items designed to evaluate alogia did not load into this dimension. Our findings showed that those items referred to poverty of content of speech, blocking and increased latency of response loaded more in inattention dimension than in expressivity although they did not load more than twice as much as other factors. On the other hand, poverty of speech loaded more in expressivity dimension but again it did not load more than twice as much as other factors. Lyne et al. (2013) found that alogia items loaded in the same factors than us ^22^. The main reason for the differences between Lyne´s findings and ours is that we have used more restrictive criteria in the choice of factors, as they did not require the loading on dimensions to be twice as high as on other factors. In addition, we have used a cut-off point of 0.50 to consider that an item loads on a factor while Lyne et al. (2013) chose 0.40. The employment of more restrictive criteria increases the reliability of the results avoiding distorted and potentially meaningless solutions ^23^.

Experiential is characterised by decreased in the outward expression of emotion and speech, including symptoms such as avolition, asociality and anhedonia ^24^. Here, this dimension was formed by items from avolition-apathy and anhedonia-asociality dimensions. Strauss et al. (2018) suggested that the division of negative symptoms into 2 dimensions does not capture the complexity of the negative symptomatology and that the latent structure is best conceptualized in relation to 5 domains (i.e., anhedonia, asociality, avolition, blunted affect and alogia) ^25^. As the authors highlight in the paper, they only included patients in the chronic illness phase and their results may not generalize to earlier phases of illness. This may be the underlying reason why two-dimensional structures have shown to fit better in early stages of the disease ^21^.

**References**

1 Pelayo-Terán JM, Pérez-Iglesias R, Ramírez-Bonilla ML, González-Blanch C, Martínez-García O, Pardo-García G, *et al.* Epidemiological factors associated with treated incidence of first-episode non-affective psychosis in Cantabria: Insights from the Clinical Programme on Early Phases of Psychosis. *Early Interv Psychiatry* 2008; **2**: 178–87.

2 Crespo-Facorro B, Gonzalez-Blanch C, Pelayo-Teran JM. *Programa asistencial para las fases iniciales de psicosis de Cantabria*. Mason, 2005.

3 Crespo-Facorro B, Pérez-Iglesias R, Ramirez-Bonilla ML, Martínez-García O, Llorca J, Vázquez-Barquero JL. A practical clinical trial comparing haloperidol, risperidone, and olanzapine for the acute treatment of first-episode nonaffective psychosis. *J Clin Psychiatry* 2006. doi:10.4088/JCP.v67n1004.

4 First MB et, Spitzer RL, Gibbon M, Williams JBW, First, Spitzer RL, *et al.* *Structured Clinical Interview for DSM-IV Axis I Disorders, Clinician Version (SCID-CV)*. American Psychiatric Press, Inc, 1997 doi:9780880489317.

5 First, Michael B., Spitzer, Robert L, Gibbon M. Structured Clinical Interview for DSM-IV Axis I Disorders, Clinician Version (SCID-CV). 1996.

6 Addington DE, McKenzie E, Norman R, Wang JL, Bond GR. Essential evidence-based components of first-episode psychosis services. *Psychiatr Serv* 2013. doi:10.1176/appi.ps.201200156.

7 González-Blanch C, Alvarez-Jiménez M, Rodríguez-Sánchez JM, Pérez-Iglesias R, Vázquez-Barquero JL, Crespo-Facorro B. Cognitive functioning in the early course of first-episode schizophrenia spectrum disorders: timing and patterns. *Eur Arch Psychiatry Clin Neurosci* 2006; **256**: 364–71.

8 Rey A. *L’examen clinique en psychologie.* , 1958 doi:CRJ Alz lib Copy2 #618; CRJ Alz lib Copy2-Converted #618; CRJ Alz Library 2 shared lib-Converted #1017; MMM Master_Reference_List-Converted #21; JLW MCI-Converted #22; JLW PCA-Converted #58; JLW VBMprediction-Converted #87.

9 Osterrieth PA. Le test de copie d’une figure complexe [in French]. *Arch Psychol (Geneve)* 1944; **30**: 206–356.

10 Reitan RM. Validity of the Trail Making Test as an Indicator or Organic Brain Damage. *Percept Mot Skills* 1958; **8**: 271–6.

11 Wechsler D. *WAIS‐III administration and scoring manual*. , 1997 doi:10.1177/1073191102009001003.

12 Lezak MD. Domains of behavior from a neuropsychological perspective: The whole story. In *Integrative views of motivation, cognition, and emotion.*: 23–55. , 1994.

13 Lezak MD. *Neuropsychological assessment (3rd ed.)*. , 1995.

14 Reichenberg A, Harvey PD, Bowie CR, Mojtabai R, Rabinowitz J, Heaton RK, *et al.* Neuropsychological function and dysfunction in schizophrenia and psychotic affective disorders. *Schizophr Bull* 2009; **35**: 1022–9.

15 O’connor BPP, O’connor BP. SPSS and SAS programs for determining the number of components using parallel analysis and Velicer’s MAP test. *Behav Res Methods, Instruments, Comput* 2000; **32**: 396–402.

16 Williams B, Brown T, Onsman A. Exploratory factor analysis : A five-step guide for novices. *J Emerg Prim Heal Care* 2012. doi:10.1080/09585190701763982.

17 Muthén LKK, Muthén BOO. Mplus User’s Guide. Eighth Edition. *Los Angeles, CA Muthén Muthén* 2017. doi:10.1111/j.1600-0447.2011.01711.x.

18 Galderisi S, Mucci A, Dollfus S, Nordentoft M, Falkai P, Kaiser S, *et al.* EPA guidance on assessment of negative symptoms in schizophrenia. *Eur Psychiatry* 2021; **64**: e23.

19 Kirkpatrick B, Fenton WS, Carpenter Jr WT, Marder SR. The NIMH-MATRICS consensus statement on negative symptoms. *Schizophr Bull* 2006; **32**: 214–9.

20 Blanchard JJ, Cohen AS. The structure of negative symptoms within schizophrenia: Implications for assessment. In *Schizophrenia Bulletin*: , 2006 doi:10.1093/schbul/sbj013.

21 Lyne J, Renwick L, Grant T, Kinsella A, McCarthy P, Malone K, *et al.* Scale for the Assessment of Negative Symptoms structure in first episode psychosis. *Psychiatry Res* 2013; **210**: 1191–7.

22 Kaiser S, Lyne J, Agartz I, Clarke M, Mørch-Johnsen L, Faerden A. Individual negative symptoms and domains – Relevance for assessment, pathomechanisms and treatment. *Schizophr Res* 2017; **186**: 39–45.

23 Watkins MW. Exploratory Factor Analysis: A Guide to Best Practice. *J Black Psychol* 2018; **44**: 219–46.

24 Kirkpatrick B, Strauss GP, Nguyen L, Fischer BA, Daniel DG, Cienfuegos A, *et al.* The brief negative symptom scale: psychometric properties. *Schizophr Bull* 2011; **37**: 300–5.

25 Strauss GP, Nuñez A, Ahmed AO, Barchard KA, Granholm E, Kirkpatrick B, *et al.* The Latent Structure of Negative Symptoms in Schizophrenia. *JAMA Psychiatry* 2018; **75**: 1271–9.
