# Supplementary material for: Longitudinal trajectories in negative symptoms and changes in brain cortical thickness: 10-year follow-up study
Source: Br J Psychiatry. 2023 Jul;223(1):309–18. doi: 10.1192/bjp.2022.192 (PMC10331319; doi:10.1192/bjp.2022.192)

## Supplementary figures

**Figure S1. Screen plot and Parallel Analysis of eigenvalues for the Scale Assessment of Negative Symptoms.**


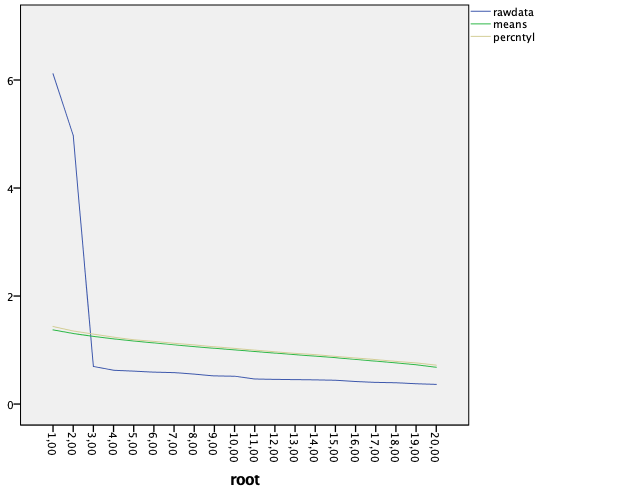


Eigenvalues

**Figure S2. Confirmatory Factor Analyses.**


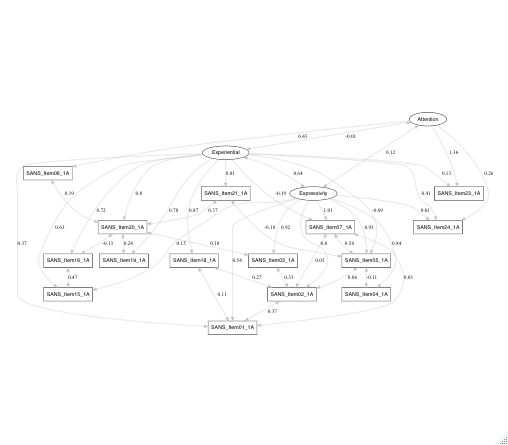


**Figure S3: Association between changes in Cortical Thickness and four clinical covariates for 10 years follow-up, including medication, cognition, positive, disorganization symptoms and attrition.**


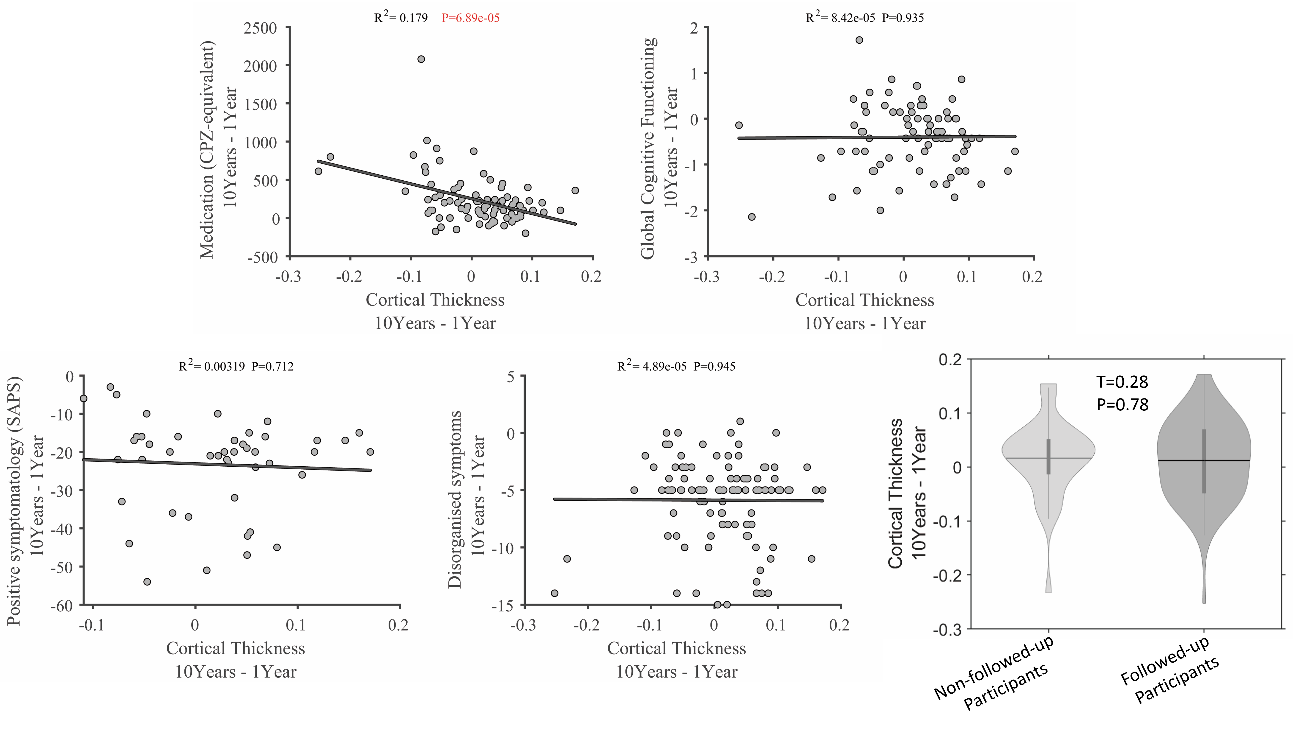


**Figure S4: Whole-brain cortical thickness changes for expressivity and experiential SANS factors after correcting for medication.**


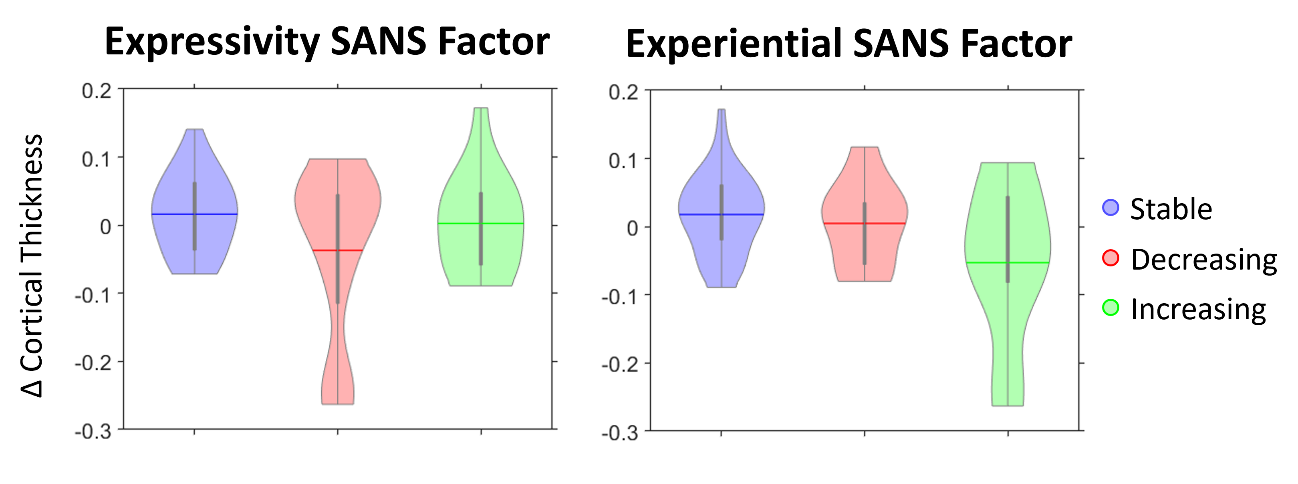


**Figure S5: Regional long-term cortical thickness changes for expressivity SANS factors after correcting for medication. Violin plots of CT longitudinal changes of regions showing a significant group effect.**


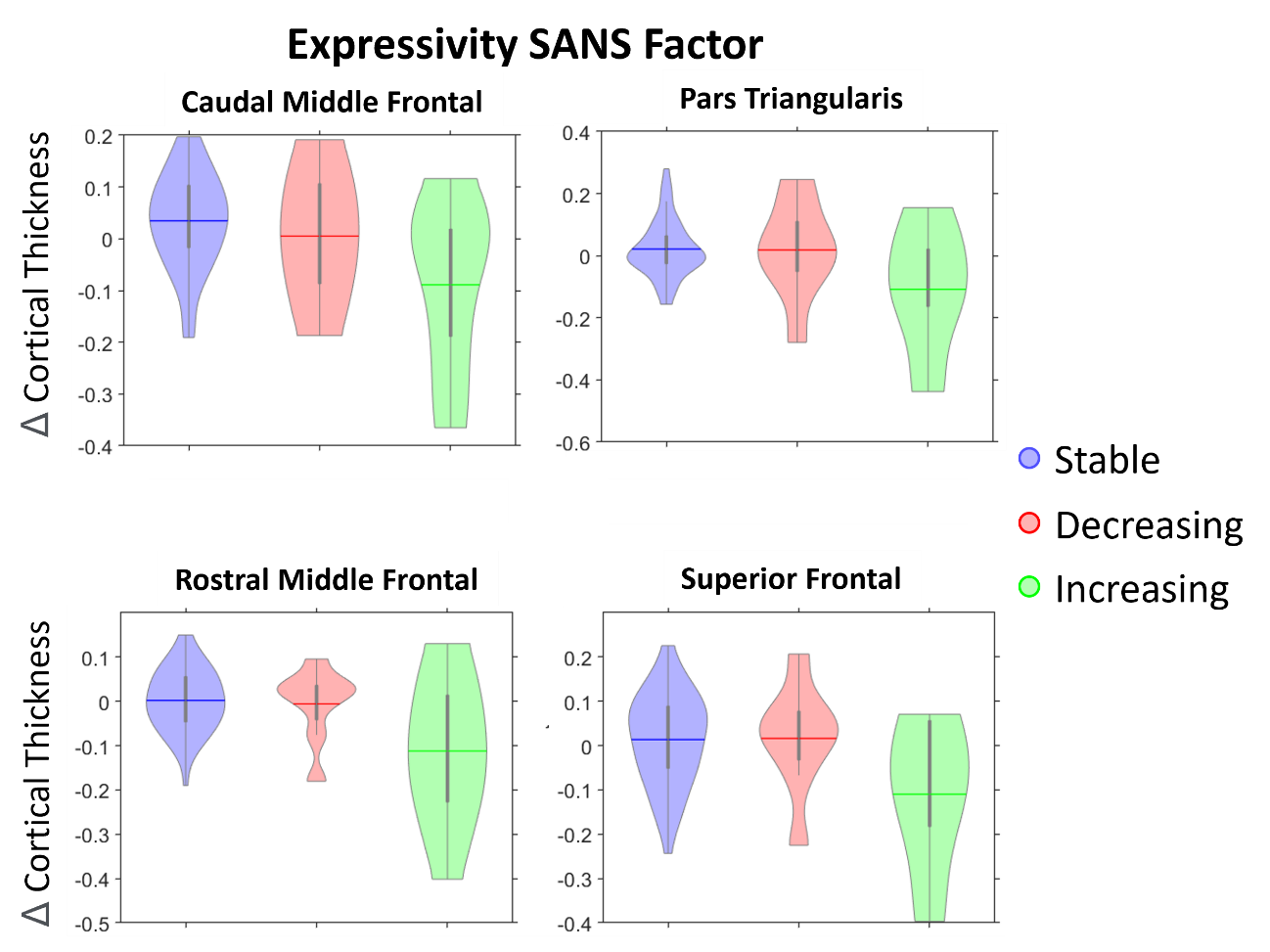

Supplement: Supplementary file 1 [file bjpsup.zip › S0007125022001921sup002.docx]
