# Supplementary material for: Longitudinal trajectories in negative symptoms and changes in brain cortical thickness: 10-year follow-up study
Source: Br J Psychiatry. 2023 Jul;223(1):309–18. doi: 10.1192/bjp.2022.192 (PMC10331319; doi:10.1192/bjp.2022.192)
Supplement: Supplementary file 1 [file bjpsup.zip › S0007125022001921sup003.docx]

| **Table 1. Exploratory Factor Analysis. Factor loadings.** | | | | | | |
| --- | --- | --- | --- | --- | --- | --- |
|  | Expressivity | Experiential | Attention | Expressivity | Experiential | Attention |
| Unchanging Facial Expression | **0.75** | 0.37 | 0.05 | **0,74** | 0,37 | 0,01 |
| Decreased Spontaneous Movements | **0.75** | 0.21 | 0.03 | **0,77** | 0,19 | 0,01 |
| Paucity of Expressive Gestures | **0.80** | 0.17 | -0.05 | **0,82** | 0,16 | -0,04 |
| Poor Eye contact | **0.78** | 0.23 | 0.12 | **0,79** | 0,23 | 0,06 |
| Affective No responsivity | **0.88** | 0.17 | -0.05 | **0,89** | 0,15 | -0,04 |
| Inappropriate Affect | -0.06 | -0.18 | **0.57** | -0,01 | -0,16 | **0,68** |
| Lack of vocal Inflections | **0.81** | 0.20 | 0.03 | **0,82** | 0,2 | 0,01 |
| Poverty of Speech | 0.64 | 0.29 | 0.38 | (-) | (-) | (-) |
| Poverty of Content of Speech | 0.41 | 0.18 | 0.52 | (-) | (-) | (-) |
| Blocking | 0.33 | 0.19 | 0.65 | (-) | (-) | (-) |
| Increased Latency of Response | 0.60 | 0.17 | 0.57 | (-) | (-) | (-) |
| Grooming and Hygiene | 0.31 | 0.57 | -0.01 | (-) | (-) | (-) |
| Impersistence at Work or School | 0.29 | **0.75** | 0.07 | 0,27 | **0,73** | -0,03 |
| Physical Anergia | 0.14 | **0.73** | 0.06 | 0,16 | **0,73** | 0,01 |
| Recreational Interest and Activities | 0.28 | **0.78** | 0.11 | 0,3 | **0,79** | 0,08 |
| Sexual Activity | 0.03 | **0.78** | 0.06 | 0,04 | **0,79** | 0,01 |
| Ability to Feel Intimacy and Closeness | 0.25 | **0.77** | 0.05 | 0,27 | **0,78** | 0,01 |
| Relationship with Friends and Peers | 0.26 | **0.81** | 0.09 | 0,27 | **0,83** | 0,03 |
| Social Inattentiveness | -0.1 | 0.16 | **0.54** | -0,04 | 0,17 | **0,66** |
| Inattentiveness During Mental Status Testing | -0.02 | 0.01 | **0.72** | 0,05 | 0,04 | **0,82** |
| **Variance explained** | 39.21% | 11.26% | 9.81% | 41.16% | 13.83% | 10.48% |
|  | 60.28% | | | 65.51% | | |
| **Kaiser-Meyer-Olkin test** | 0.90 | | | 0.88 | | |
| **Bartlett´s sphericity test** | 3577.24 (p<.001) | | | 2565.28 (p<.001) | | |
